# Time-Resolved Three-Dimensional Contrast-Enhanced Magnetic Resonance Angiography in Patients with Chronic Expanding and Stable Aortic Dissections

**DOI:** 10.1155/2017/5428914

**Published:** 2017-11-28

**Authors:** Michael Trojan, Fabian Rengier, Drosos Kotelis, Matthias Müller-Eschner, Sasan Partovi, Christian Fink, Christof Karmonik, Dittmar Böckler, Hans-Ulrich Kauczor, Hendrik von Tengg-Kobligk

**Affiliations:** ^1^Department of Diagnostic and Interventional Radiology, University Hospital Heidelberg, Im Neuenheimer Feld 110, 69120 Heidelberg, Germany; ^2^Department of Radiology, German Cancer Research Center (DKFZ), Im Neuenheimer Feld 280, 69120 Heidelberg, Germany; ^3^Department of Vascular and Endovascular Surgery, University Hospital Heidelberg, Im Neuenheimer Feld 110, 69120 Heidelberg, Germany; ^4^Department of Radiology, University Hospitals Cleveland Medical Center, Case Western Reserve University, 11100 Euclid Ave, Cleveland, OH, USA; ^5^Department of Radiology, AKH Celle, Siemensplatz 4, 29223 Celle, Germany; ^6^MRI Core, Houston Methodist Research Institute, Houston, TX, USA; ^7^Department for Diagnostic, Interventional and Paediatric Radiology, University Hospital Bern, Inselspital, University of Bern, Freiburgstr. 10, 3010 Bern, Switzerland

## Abstract

**Objective:**

To prospectively evaluate our hypothesis that three-dimensional time-resolved contrast-enhanced magnetic resonance angiography (TR-MRA) is able to detect hemodynamic alterations in patients with chronic expanding aortic dissection compared to stable aortic dissections.

**Materials and Methods:**

20 patients with chronic or residual aortic dissection in the descending aorta and patent false lumen underwent TR-MRA of the aorta at 1.5 T and repeated follow-up imaging (mean follow-up 5.4 years). 7 patients showed chronic aortic expansion and 13 patients had stable aortic diameters. Regions of interest were placed in the nondissected ascending aorta and the false lumen of the descending aorta at the level of the diaphragm (FL-diaphragm level) resulting in respective time-intensity curves.

**Results:**

For the FL-diaphragm level, time-to-peak intensity and full width at half maximum were significantly shorter in the expansion group compared to the stable group (*p* = 0.027 and *p* = 0.003), and upward and downward slopes of time-intensity curves were significantly steeper (*p* = 0.015 and *p* = 0.005). The delay of peak intensity in the FL-diaphragm level compared to the nondissected ascending aorta was significantly shorter in the expansion group compared to the stable group (*p* = 0.01).

**Conclusions:**

3D TR-MRA detects significant alterations of hemodynamics within the patent false lumen of chronic expanding aortic dissections compared to stable aortic dissections.

## 1. Introduction

Chronic expansion of aortic dissection is a frequent complication in patients with aortic dissection [1–3]. It occurs both in uncomplicated Stanford type B aortic dissection on best medical treatment and in Stanford type A or type B aortic dissection after operation or intervention. Increasing aortic diameters carry the risk of aortic rupture as a potentially fatal event [4]. Thus, patients with aortic dissection require life-long imaging follow-up to detect potential aortic expansion which can be treated by endovascular repair or surgical procedures [5, 6].

The interval for imaging surveillance is relevant both in terms of radiation exposure when using computed tomography (CT) as it is frequently the case and in terms of healthcare cost. Up to date, imaging intervals are standardized for all patients with aortic dissection usually without considering individual risk factors for aortic expansion. It would be desirable to estimate the individual risk for aortic expansion to determine the individually optimal imaging interval, to depict the time point of intervention if necessary, and ultimately to prevent potentially fatal aortic rupture.

Previous investigations focused on clinical and morphological parameters for risk stratification of patients with aortic dissection. Parameters suggested to be associated with higher risk of aortic expansion include patient age, total aortic diameter, false lumen diameter, greater primary entry tear diameter, primary entry tear location, and false lumen thrombosis [1, 5, 7–10]. Little research has been performed regarding functional or hemodynamic parameters in aortic dissection although the potential of functional imaging techniques to advance understanding of pathophysiological mechanisms and to risk stratify patients has been repeatedly stated [11]. Early studies reported differences of blood flow in the false lumen compared to the true lumen by using 2D velocity-encoded magnetic resonance imaging (MRI) [12]. Later studies using 2D velocity-encoded MRI demonstrated different patterns of blood flow within the false lumen and showed potential association between those patterns and aortic expansion [13, 14].

Time-resolved contrast-enhanced magnetic resonance angiography (TR-MRA) has been applied to aortic dissection for visual assessment of dissection morphology and blood flow [15–17]. The technique, however, also offers the possibility for quantitative evaluation of hemodynamics similar to the arterial input function in dynamic contrast-enhanced MRI [18–20]. Whereas 2D and 4D flow MRI measure blood velocity and provide information on only one single cardiac cycle by repeated acquisitions over several cardiac cycles, TR-MRA dynamically measures contrast media dispersion with the blood stream over a period of several cardiac cycles. To our knowledge, TR-MRA has not yet been investigated with respect to hemodynamics in aortic dissection and chronic expansion of aortic dissection.

The aim of this study was to prospectively evaluate our hypothesis that three-dimensional TR-MRA is able to detect hemodynamic alterations in patients with chronic expanding aortic dissection compared to stable aortic dissection.

## 2. Materials and Methods

### 2.1. Subjects

This prospective study was approved by the institutional review board and written informed consent was obtained prior to enrolment into the study. 26 patients with known aortic dissection currently on best medical treatment agreed to undergo an MRI acquisition in addition to a clinically indicated CT scan. Exclusion criteria for participating in the study were emergency situations, contraindications for performing an MRI scan (e.g., cardiac pacemaker), impaired renal function, previous allergic reactions to Gadolinium, claustrophobia, and pregnancy. Six patients were secondarily excluded due to complete false lumen thrombosis at initial MRI (*n* = 4) or loss to follow-up (*n* = 2). The final study population consisted of 20 patients with aortic dissection (mean age 65 ± 9 years, age range 49–78, 5 female, 15 male), thereof 11 patients with uncomplicated aortic dissection type Stanford B without prior aortic surgery, 2 patients with aortic dissection type Stanford B with prior aortic surgery and residual distal aortic dissection (1 interposition tube graft in the proximal descending aorta and 1 thoracic endovascular aortic repair), and 7 patients with residual aortic dissection in the descending aorta after ascending aortic replacement due to aortic dissection type Stanford A.

### 2.2. Time-Resolved Contrast-Enhanced Magnetic Resonance Angiography

**Figure 1:**
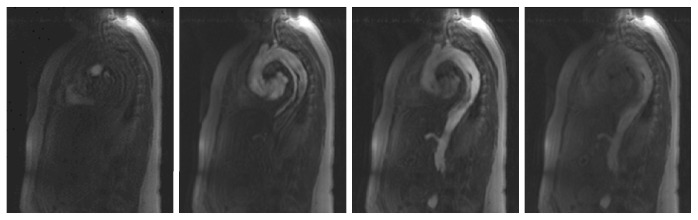
Representative time steps of a time-resolved MRA data set in a patient with aortic dissection Stanford type B. Note the gradual filling of the true lumen (anterior) and the false lumen (posterior) with the contrast medium followed by decrease of the intensity in both lumina.

A 1.5 T clinical MR scanner (Magnetom Symphony, Siemens, Erlangen, Germany) and two standard phased array body coils were used for MRI acquisition. Patients were positioned in supine position. For TR-MRA, a time-resolved three-dimensional gradient echo pulse sequence with view sharing (time-resolved angiography with stochastic trajectories, TWIST) was applied in sagittal orientation (Figure 1). The following sequence parameters were used: field of view 500 × 325 mm; in-plane resolution, 2.6 × 2.6 mm; slice thickness, 3 mm; 30 slices per slab; parallel imaging GRAPPA, PAT factor 2; repetition/echo time (TR/TE), 1.82/0.68 ms; flip angle, 40°; acquisitions every 2.4 s over a period of 2 min. Acquisition was performed during shallow breathing. Gadolinium contrast agent (Gd-DTPA, Magnevist, Bayer Schering, Germany) was administered with a dose of 0.1 mmol/kg body weight via an 18 G cannula in an antecubital vein with an injection rate of 3 ml/s followed by a saline flush of 20 ml at the same injection rate using an automatic power injector.

### 2.3. Image Analysis

**Figure 2:**
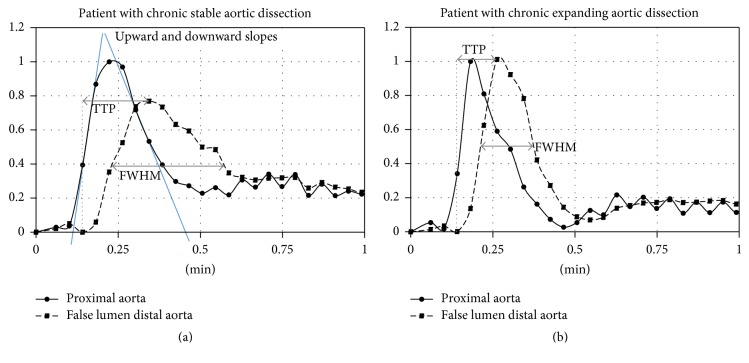
Time-intensity curves as measured by time-resolved MRA in representative patients with chronic expanding aortic dissection (a) and chronic stable aortic dissection (b). *x*-axis is plotted in minutes; *y*-axis is dimensionless with 1 representing peak enhancement. For each patient, two curves are shown. The continuous line represents the intensity in the nondissected proximal aorta; the dashed line represents the intensity in the false lumen at the level of the diaphragm. Time to peak (TTP) and full width at half maximum (FWHM) are illustrated for the dashed curve. Upward and downward slopes (in blue) are visualized for the continuous line on the left.

MRI data was transferred to Syngo MultiModality Workplace VE36A (Siemens, Erlangen, Germany). All data was analyzed by one radiologist experienced in cardiovascular imaging. Regions of interest were placed in the lumen of the nondissected distal ascending aorta just proximal the aortic arch and of the true and false lumen of the descending aorta at the level of the diaphragm covering as much of the respective lumen as possible. From the resulting time-intensity-curves, the following parameters were computed as it has been described in literature (Figure 2) [18, 21]: time-to-peak (TTP) intensity, full width at half maximum (FWHM), and upward slope and downward slope. Furthermore, the difference between the time step with peak intensity in the false lumen and the time step with peak intensity in the nondissected proximal aorta was calculated.

### 2.4. Follow-Up and Aortic Expansion

All patients also underwent CT at the time of the initial MRI. Patients then received regular follow-up in our outpatient clinic and further CT or MRI scans were acquired as clinically indicated with a mean imaging follow-up period of 5.4 ± 3.7 years (range 1.2–11.2 years). For each patient, maximum diameters of the thoracic and the abdominal aorta were measured at identical positions on the initial scan and on all imaging follow-ups. These measurements were performed by a different radiologist experienced in cardiovascular imaging who was not involved in reading the TR-MRA data to avoid potential bias. Patients were assigned to the expansion group if both of the following criteria were fulfilled: an increase of either thoracic or abdominal aortic diameter over at least three consecutive exams within a period of three years and an increase of aortic diameter of at least 6 mm. This definition was chosen to identify patients above the mean expansion rate of 1.7 mm/year shown by data from the International Registry of Acute Aortic Dissection (IRAD) and beyond any measurement errors [22].

### 2.5. Statistical Analysis

Data is given as mean ± standard deviation. Wilcoxon's signed rank test or Chi-square test was applied as appropriate to test for differences between groups for ratio-scaled or nominal-scaled data, respectively. A *p* value of ≤0.05 was considered to represent statistical significance. All analyses were performed with SPSS Version 23.0 (SPSS Inc., Chicago, IL, USA).

## 3. Results

During the follow-up, 7 patients fulfilled the criteria of aortic expansion. The remaining 13 patients served as control group with stable aortic diameter during follow-up. Distribution of age, gender, type of dissection, and proportion of prior aortic surgery were similar between the two groups (Table 1). None of the patients showed complete false lumen thrombosis during imaging follow-up.

In the nondissected proximal aorta, TTP, FWHM, and upward slope and downward slope of time-intensity curves did not significantly differ between the two groups. The respective values were for the expansion/stable group: TTP [s] 7.8 ± 2.2/8.7 ± 1.9 (*p* = 0.43), FWHM [s] 10.5 ± 4.0/11.4 ± 2.1 (*p* = 0.38), upward slope 12.2 ± 9.0/12.8 ± 2.9 (*p* = 1.0), and downward slope −5.4 ± 4.4/−6.2 ± 2.5 (*p* = 1.0). For the descending aorta at the level of the diaphragm, significant differences between the expansion group and the stable group were observed in the false lumen but not in the true lumen (Table 2). Peak intensity in the false lumen of the descending aorta at the level of the diaphragm occurred significantly faster in the expansion group compared to the stable group but not in the true lumen (Table 2).

For the expansion group, none of the time-intensity curve characteristics significantly differed between the false lumen and the true lumen at the level of the diaphragm. For the stable group, there were significant differences between the false lumen and the true lumen of the aortic dissection at the level of the diaphragm regarding FWHM (*p* = 0.013), upward slope (*p* = 0.013), downward slope (*p* = 0.013), and peak intensity delay (*p* = 0.008).

## 4. Discussion

To our knowledge, this is the first study investigating TR-MRA with respect to quantification of aortic dissection hemodynamics and chronic expansion of aortic dissection. TR-MRA demonstrated significant differences of contrast dynamics between patients with chronic expanding and patients with stable aortic dissection. Patients with chronic expansion of aortic dissection exhibited significantly shorter TTP and FWHM and significantly steeper upward and downward slopes of time-intensity curves in the patent false lumen of the aortic dissection at the level of the diaphragm compared to patients with stable aortic dissection. Moreover, the time interval between peak intensities in the nondissected proximal aorta and the patent false lumen at the level of the diaphragm was significantly shorter in patients with chronic expanding aortic dissection (CEAD). On the other hand, no significant differences were observed for contrast characteristics in the nondissected proximal aorta and in the true lumen at the level of the diaphragm.

Considering that time-intensity curves did not significantly differ between the expansion and the stable group at the level of the nondissected proximal aorta and in the true lumen at the level of the diaphragm, we conclude that hemodynamic changes in patients with expanding aortic dissection and patent false lumen mainly occur within the false lumen. Within the false lumen, patients with chronic expanding aortic dissection showed steeper rather skewed time-intensity curves as expressed by the short FWHM and higher upward and downward slopes, with very similar characteristics to time-intensity curves of the true lumen. On the contrary, patients with stable aortic dissection exhibited flatter, wider time-intensity curves within the false lumen with significant differences to time-intensity curves of the true lumen.

These findings are well in accordance with findings by Inoue et al. who observed increased expansion in patients with higher antegrade flow in the false lumen as measured by 2D velocity-encoded MRI [14]. In TR-MRA, higher antegrade flow in the false lumen translates to faster filling with contrast as well as faster washout of contrast, the pattern that was also associated with expansion in our study. Other patterns of flow within the false lumen described by previous studies applying 2D velocity-encoded MRI are retrograde or bidirectional flow [12–14]. In the study by Inoue et al., these patterns were associated with stable aortic dissection [14]. In TR-MRA, retrograde or bidirectional flow in the false lumen will lead to slower filling with contrast and slower washout of contrast, the pattern that was also associated with stable disease in our study.

Higher antegrade flow in the false lumen could be explained by an accelerated inflow into the false lumen. Potential hemodynamic mechanisms of accelerated inflow into the false lumen are complex and likely multifactorial, making an evaluation by a single morphological measure difficult. A larger primary entry tear, a main blood stream pointed more directly towards the primary entry tear, several proximally located reentries, false lumen size, or increased outflow from the false lumen to the iliac arteries or abdominal aortic branches might all contribute to an accelerated inflow into the false lumen. Several of these factors have been shown to be associated with expansion of aortic dissection [1, 5, 7–10]. Hence hemodynamics within the aortic dissection might be a more direct measure of pathophysiological processes than morphology alone. Other methods for assessing hemodynamics within aortic dissections have been evaluated in pilot studies, in particular 2D and 4D flow MRI and computational fluid dynamics [11, 12, 23]. Whereas 2D and 4D flow MRI measure blood velocity and provide information on only one single cardiac cycle by repeated acquisitions over several cardiac cycles, TR-MRA dynamically measures contrast media dispersion with the blood stream over a period of several cardiac cycles. Therefore, these techniques may provide complimentary information.

This study has some limitations. First of all, the study population was relatively small. However, the primary purpose of the present study was to demonstrate the feasibility and the potential of TR-MRA in patients with chronic aortic dissection, justifying a subsequent study in a larger patient population. Second, it might be criticized that the study population included patients with both Stanford type A and Stanford type B dissections. However, the number of patients with each type of dissection did not significantly differ between the expansion group and the stable group. Moreover, patients with both types of dissection exhibited the same characteristics of hemodynamics as assessed by TR-MRA when expanding or staying stable, respectively. Third, this study did not investigate the relationship of hemodynamics to distal tears. Finally, assessment of scan-rescan variability was not part of this study because we wanted to avoid repeated contrast medium administration within a short interval. Reproducibility of free-breathing TR-MRA has been demonstrated previously [24].

## 5. Conclusions

Three-dimensional TR-MRA detects significant alterations of contrast dynamics within the patent false lumen of chronic expanding aortic dissections compared to stable aortic dissections which may be explained by an accelerated inflow into and outflow from the false lumen. The potential of the technique for risk assessment of aortic expansion needs to be studied in larger patient populations.

## Figures and Tables

**Table 1:** Clinical characteristics for both patient groups.

	Group with chronic expanding aortic dissection (*n* = 7)	Group with chronic stable aortic dissection (*n* = 13)	*p* value
Age	65 ± 8 (53–78)	64 ± 9 (49–78)	0.94
Gender	5 male, 2 female	10 male, 3 female	0.79
Type of dissection	5 Stanford type B, 2 Stanford type A	8 Stanford type B, 5 Stanford type A	0.66
Prior aortic surgery	4 without prior surgery, 3 with prior surgery	7 without prior surgery, 6 with prior surgery	0.74

**Table 2:** Time-intensity curve characteristics of the aortic dissection at the level of the diaphragm.

Lumen	False lumen			True lumen		
Group	Expansion	Stable	*p* value	Expansion	Stable	*p* value
TTP [s]	8.4 ± 3.4	13.2 ± 5.5	0.027	8.4 ± 2.7	9.6 ± 3.6	0.44
FWHM [s]	10.8 ± 2.2	24.7 ± 10.2^*∗*^	0.003	10.7 ± 3.8	13.0 ± 6.4	0.40
Upward slope	13.5 ± 6.9	7.6 ± 2.7^*∗*^	0.015	13.4 ± 2.8	11.8 ± 3.5	0.25
Downward slope	−7.0 ± 1.4	−2.0 ± 2.7^*∗*^	0.005	−5.0 ± 3.0	−6.2 ± 3.0	0.66
Delay of peak intensity compared to nondissected aorta [s]	3.0 ± 4.3	10.1 ± 6.4^*∗*^	0.01	1.8 ± 1.5	2.7 ± 4.0	0.66

^*∗*^Significant differences (*p* < 0.05) between the false lumen and the true lumen for the stable group only.

## References

[1] Song J.-M., Kim S.-D., Kim J.-H. (2007). Long-Term Predictors of Descending Aorta Aneurysmal Change in Patients With Aortic Dissection. *Journal of the American College of Cardiology*.

[2] Luebke T., Brunkwall J. (2014). Type B Aortic Dissection: A Review of Prognostic Factors and Meta-analysis of Treatment Options. *Aorta Stamford Conn*.

[3] Malvindi P. G., Van Putte B. P., Sonker U., Heijmen R. H., Schepens M. A. A. M., Morshuis W. J. (2013). Reoperation after acute type A aortic dissection repair: A series of 104 patients. *The Annals of Thoracic Surgery*.

[4] Davies R. R., Goldstein L. J., Coady M. A. (2002). Yearly rupture or dissection rates for thoracic aortic aneurysms: Simple prediction based on size. *The Annals of Thoracic Surgery*.

[5] Trimarchi S., Jonker F. H. W., van Bogerijen G. W. H. (2014). Predicting aortic enlargement in type B aortic dissection. *Ann Cardiothorac Surg*.

[6] Nienaber C. A., Divchev D., Palisch H., Clough R. E., Richartz B. (2014). Early and late management of type B aortic dissection. *Heart*.

[7] Evangelista A., Salas A., Ribera A. (2012). Long-term outcome of aortic dissection with patent false lumen: Predictive role of entry tear size and location. *Circulation*.

[8] Kamman A. V., Brunkwall J., Verhoeven E. L. (2017). Predictors of aortic growth in uncomplicated type B aortic dissection from the Acute Dissection Stent Grafting or Best Medical Treatment (ADSORB) database. *Journal of Vascular Surgery*.

[9] Weiss G., Wolner I., Folkmann S. (2012). The location of the primary entry tear in acute type B aortic dissection affects early outcome. *European Journal of Cardio-Thoracic Surgery*.

[10] Kotelis D., Grebe G., Kraus P. (2016). Morphologic predictors of aortic expansion in chronic type B aortic dissection. *Vascular*.

[11] Clough R. E., Zymvragoudakis V. E., Biasi L. (2014). Usefulness of new imaging methods for assessment of type B aortic dissection. *Ann Cardiothorac Surg*.

[12] Chang J.-M., Friese K., Caputo G. R., Kondo C., Higgins C. B. (1991). Mr measurement of blood flow in the true and false channel in chronic aortic dissection. *Journal of Computer Assisted Tomography*.

[13] Strotzer M., Aebert H., Lenhart M. (2000). Morphology and hemodynamics in dissection of the descending aorta: Assessment with MR imaging. *Acta Radiologica*.

[14] Inoue T., Watanabe S., Sakurada H. (2000). Evaluation of flow volume and flow patterns in the patent false lumen of chronic aortic dissections using velocity-encoded cine magnetic resonance imaging. *Japanese Circulation Journal*.

[15] Kinner S., Eggebrecht H., Maderwald S. (2015). Dynamic MR angiography in acute aortic dissection. *Journal of Magnetic Resonance Imaging*.

[16] Krishnam M. S., Tomasian A., Lohan D. G., Tran L., Finn J. P., Ruehm S. G. (2008). Low-dose, time-resolved, contrast-enhanced 3D MR angiography in cardiac and vascular diseases: correlation to high spatial resolution 3D contrast-enhanced MRA. *Clinical Radiology*.

[17] Schoenberg S. O., Wunsch C., Knopp M. V. (1999). Abdominal aortic aneurysm: Detection of multilevel vascular pathology by time-resolved multiphase 3D gadolinium MR angiography: Initial report. *Investigative Radiology*.

[18] Gordon Y., Partovi S., Müller-Eschner M. (2014). Dynamic contrast-enhanced magnetic resonance imaging: fundamentals and application to the evaluation of the peripheral perfusion. *Cardiovasc Diagn Ther*.

[19] Isbell D. C., Epstein F. H., Zhong X. (2007). Calf muscle perfusion at peak exercise in peripheral arterial disease: Measurement by first-pass contrast-enhanced magnetic resonance imaging. *Journal of Magnetic Resonance Imaging*.

[20] Haeck J., Bol K., Bison S. (2015). Optimized time-resolved imaging of contrast kinetics (TRICKS) in dynamic contrast-enhanced MRI after peptide receptor radionuclide therapy in small animal tumor models. *Contrast Media & Molecular Imaging*.

[21] Mendichovszky I. A., Cutajar M., Gordon I. (2009). Reproducibility of the aortic input function (AIF) derived from dynamic contrast-enhanced magnetic resonance imaging (DCE-MRI) of the kidneys in a volunteer study. *European Journal of Radiology*.

[22] Jonker F. H. W., Trimarchi S., Rampoldi V. (2012). Aortic expansion after acute type B aortic dissection. *The Annals of Thoracic Surgery*.

[23] Cheng Z., Juli C., Wood N. B., Gibbs R. G. J., Xu X. Y. (2014). Predicting flow in aortic dissection: Comparison of computational model with PC-MRI velocity measurements. *Medical Engineering & Physics*.

[24] Ingrisch M., Maxien D., Schwab F., Reiser M. F., Nikolaou K., Dietrich O. (2014). Assessment of pulmonary perfusion with breath-hold and free-breathing dynamic contrast-enhanced magnetic resonance imaging: Quantification and reproducibility. *Investigative Radiology*.

